# Persistent psychotic symptoms following COVID-19 infection

**DOI:** 10.1192/bjo.2020.76

**Published:** 2020-07-22

**Authors:** Soon Tjin Lim, Benjamin Janaway, Harry Costello, Anand Trip, Gary Price

**Affiliations:** Department of Neurology, University College Hospital, UCLH, UK; Department of Neuropsychiatry, National Hospital for Neurology and Neurosurgery, UCLH, UK; Department of Neuropsychiatry, National Hospital for Neurology and Neurosurgery, UCLH, UK; Queen Square Multiple Sclerosis Centre, National Hospital for Neurology and Neurosurgery, UCLH, UK; Department of Neuropsychiatry, National Hospital for Neurology and Neurosurgery, UCLH, UK

**Keywords:** Psychotic disorders, schizophrenia, clinical neurology, cognitive neuroscience, neuroimmunology

## Abstract

To date, there have been no detailed reports of patients developing persistent psychotic symptoms following Coronavirus disease 2019 (COVID-19) infection. There have been reports of patients developing transient delirium (with and without hypoxia) after COVID-19 infection as well as other neurological manifestations. We report on a female patient who, post-COVID-19 infection, developed an initial delirium followed by persistent and florid psychotic symptoms consisting of persecutory delusion, complex visual and auditory hallucinations and Capgras phenomenon in the absence of hypoxia but elevated tumour necrosis factor (TNF)-α. The psychotic symptoms persisted for about 40 days. Her magnetic resonance imaging brain scan, electroencephalogram, cerebrospinal fluid examination and extensive autoimmune panel did not show any abnormalities. The cause of the psychotic symptoms in this patient were not ascertained but we propose either an inflammatory state, characterised by the patient's elevated TNF-alpha levels as a possible contributing mechanism for her psychosis in line with the proinflammatory changes observed in some cases of psychosis. Or, an alternative, but unproven, hypothesis is one of an antibody-mediated encephalitic event induced by viral infection.

Coronavirus disease 2019 (COVID-19) is caused by severe acute respiratory syndrome, coronavirus 2 (SARS-CoV-2). It has had a major impact on human morbidity and mortality as well as significant economic effects as it has reached a pandemic level. Increased anxiety and stress in non-infected individuals have been reported.^1^ In addition, reported neurological manifestations in infected individuals include stroke, Guillain–Barré syndrome, encephalopathy, encephalitis, epilepsy and others.^2–5^ Various immunological findings in patients with COVID-19 have been recorded including lymphopenia, raised C-reactive protein (CRP) and raised proinflammatory cytokines such as tumour necrosis factor (TNF)-alpha and interleukin (IL)-6.^6^ Aside from COVID-19, these have been associated with encephalopathic states.^7^ Psychotic symptoms have been reported in patients with COVID-19 infection,^8^ but no cases of patients with persistent psychotic symptoms have been reported in detail.

An inflammatory syndrome has been postulated as a cause for emergent psychosis in schizophrenia and schizophreniform psychosis.^9,10^ Reports linking viral infections and subsequent psychosis have dated as far back as the eighteenth century with acute ‘psychoses of influenza’ described during multiple pandemics. Raised levels of TNF and other cytokines have been observed in patients with first-episode psychosis supporting this hypothesis.^11^ The exact mechanism for the relationship is, however, unknown.

## Method

We describe the demographic, clinical, laboratory, neurophysiological and neuroradiological characteristics of a patient with proven COVID-19 infection who initially improved after developing characteristic COVID-19 symptoms. She then developed a delirium followed by persistent psychotic symptoms in the absence of delirium.

## Results

A 55-year-old White woman was admitted with a 14-day history of fever, myalgia, cough, breathlessness, loss of sense of smell and taste, and headache. Prior to this presentation she was fit and well, was on no regular medications and her only past medical history consisted of resolved renal calculi. She had no history of mental illness and there was no family history of neurological or psychiatric conditions.

On initial presentation, oxygen saturation was 94% on room air. Chest X-ray showed bilateral lower zone and peripheral pulmonary infiltrates. Chest computed tomography (CT) showed bilateral subpleural ill-defined ground-glass opacities and interlobular septal thickening in keeping with COVID-19. Nasopharyngeal swab was positive for COVID-19 infection. She received intravenous fluids, was given oxygen via nasal cannulae, which was quickly weaned. She received no other treatment and was discharged home well after 2 days.

The following day, she was confused with odd behaviour and was again admitted to hospital via ambulance. She had florid visual hallucinations of animals where she thought her cat was a lion and saw ‘monkeys jumping out of the paramedic's bag’. There were no reported automatisms, tremors or seizures.

Observations during the second hospital admission were normal, with oxygen saturation of 98% on room air. On admission to the emergency department she was disoriented to time and place, forgetting the access code to her phone, and stating that she was ‘in heaven’. She became agitated and aggressive towards staff, spitting and throwing items of clothing and swearing. She required intramuscular lorazepam to manage agitation.

She was admitted to a medical ward and appeared anxious and suspicious of staff. She described paranoid delusions involving colour symbolism whereby she attributed the colour red to people who were trying to kill her. She believed that the nursing staff were ‘devils’, trying to harm her and a family member. She exhibited bizarre behaviours including washing her phone in the sink, and repetitively brushing her teeth with soapy water. Lorazepam was used to manage acute periods of agitation and sleep was significantly disrupted. Her level of agitation and confusion continued to fluctuate. She remained disoriented to time and place but had relative periods of lucidity where she was able to have telephone conversations with her spouse.

Insight into these paranoid beliefs and agitation appeared to fluctuate, at times becoming tearful when remembering her aggression towards staff. She consistently described her mood as ‘worried’ and had a labile affect. On day 5 of admission her agitation increased again. She had persecutory delusions of a family member being murdered by staff. Haloperidol 0.5 mg twice a day was commenced 1 week into admission. She became less agitated and sleep improved, but she remained tearful, felt scared at times and appeared guarded. Oral intake was reduced and required prompting by nursing staff.

On day 10 of admission she was admitted to a neuropsychiatry ward. On admission, she appeared well kempt and made good eye contact. She was by now oriented in time and place. She was distracted at times, muttered to herself at night and appeared suspicious of certain staff members. Her speech was normal in tone, rate and rhythm. She again described her mood as ‘worried’ and had a labile affect, at times appearing elated and asking staff to dance to music on the radio, but then becoming tearful.

She continued to disclose feeling anxious about the colour red and believed it meant she was at risk of being harmed by staff, but demonstrated fluctuating insight into this. She described a Capgras-like delusion regarding a family member looking and sounding the same but having been replaced by someone else, when interacting with them over a video call. She denied any further visual hallucinations but experienced third-person persecutory auditory hallucinations and a ‘chopping sound’. She later reported a delusional idea that a family member had been admitted to the hospital.

Laboratory investigations (summarised in Tables 1 and 2) demonstrated the characteristic haematological changes of COVID-19, including pro-coagulopathic states and a transient lymphopenia.

**Table 1:** Blood parameters during hospital admission

Test	19 April	20 April	22 April	23 April	26 April	27 April	28 April	30 April	4 May
White cell count (3–10 × 10^9^/L)	6.41	4.27	6.51	–	6.10	6.58	7.19	7.85	6.35
Haemoglobin (115–155 g/L)	124	122	113	–	120	127	132	137	121
Mean corpuscular volume (80–99 fL)	84.4	87.4	85	–	85	86	85.8	88	88.1
Platelet count (150–400 × 10^9^/L)	316	312	454	–	509	544	642	542	318
Neutrophils (2–7.5 × 10^9^/L)	4.4	3.19	4.26	–	4.18	4.43	5.2	5.85	3.92
Lymphocytes (1.2–3.65 × 10^9^/L)	1.28	1.09	1.42	–	1.17	1.18	1.17	1.33	1.55
Monocytes (0.2–1 × 10^9^/L)	0.58	0.48	0.61	–	0.59	0.65	0.77	0.62	0.74
Eosinophils (0–0.4 × 10^9^/L)	0.13	0.16	0.19	–	0.12	0.28	0.01	0.01	0.10
Basophils (0–0.1 × 10^9^/L)	0.02	0.02	0.03	–	0.03	0.03	0.04	0.05	0.04
Sodium (135–145 mmol/L)	141	142	140	140	142	–	–	144	140
Potassium (3.5–5.1 mmol/L)	3.6	4.5	–	3.8	3.7	–	–	3.6	4.4
Urea (mmol/L)	–	–	–	3	–	–	–		
Creatinine (49–92 mmol/L)	71	55	57	51	59	–	–	68	66
Estimated GFR (>90 mL/min/1.73 m^2^)	78	>90	>90	>90	>90	–	–	82	86
Bilirubin (total) (0–20 μmol/L)	8	7	7	–	8	–	–	14	8
Alkaline phosphatase (35–104 IU/L)	79	75	70	82	–	–	90	90	
Alanine aminotransferase (10–35 IU/L)	29	24	–	141	–	–	123	101	
Albumin (34–50 IU/L)	40	37	39	35	42	–	–	48	45
Calcium (2.2–2.6 mmol/L)	–	2.05	–	2.11	–	–	–	2.46	–
Corrected calcium (2.2–2.6 mmol/L)	–	2.21	–	2.31	–	–	–	2.41	–
Phosphate (0.87–1.45 mmol/L)	–	0.94	–	1.27	–	–	–	1.04	–
C-reactive protein; (0–5 mg/L)	121.2	–	37.9	–	10.7	–	6.1	3.8	–
Prothrombin time (10–12 s)	11.1	–	13.9	–	–	11.6	–	–	–
International normalised ratio	1	–	1.27	–	–	1.05	–	–	–
Activated partial thromboplastin time (25–37 s)	31	–	46	–	–	32	–	–	–
Activated partial thromboplastin time ratio (0.8–1.2)	1	–	1.5	–	–	1	–	–	–
Blood culture	No growth								
COVID-19 swab	Positive			Negative					
D-dimer (0–550 μg/L FEU)	1200	–	–	–	–	–	–	–	990
Fibrinogen (135–214 IU/L)	7.28	–	–	–	–	–	–	–	
Lactate									
Dehydrogenase (135–214 IU/LIU/L)	344	–	–	–	–	–	–	–	–
Ferritin (13–150 μg/L)	1291	–	–	–	–	–	–	–	–
Tumour necrosis factor-alpha (0.10–1.75 pg/mL)	6.47	–	–	–	–	–	–	2.01	–

GFR, glomerular filtration rate.

**Table 2:** Other laboratory parameters measured in the patient during admission

Laboratory parameters	Result	Normal range
**Blood**		
*NMDA antibodies*	Negative	-
*CASPR2 antibodies*	Negative	-
*LGI1 antibodies*	Negative	-
IL1-b	0.22 pg/mL	0.11–24.30 pg/mL
IL6	3.68 pg/mL	0.16–27.20 pg/mL
IL10	0.91 pg/mL	0.06–3.08 pg/mL
Paraneoplastic screen *(Purkinje Cells, Anti Tr, Other Cerebellar cells, IgG White Matter (myelin), anti-Hu, anti-Yo, anti-Ri)*	Negative	-
HIV 1+ 2	Negative	-
TSH	3.41 mIU/l	0.27–4.20 mIU/L
T4	16.8 pmol/L	12.0–22.0 pmol/L
Prolactin	503^a^	102–496 mIU/L
B12	914 pg/ml^a^	197–771 pg/mL
Syphilis	Negative	-
Blood group	Apos	-
Troponin-T	5	0–14 ng/L
Triglyceride	2.3 mmol/L	0.4–2.3 mmol/L
Folate	5 ng/ml	2.9–26.8 ng/mL
Glucose	5.3 mmol/L	3.9–5.8 mmol/L
Drug Screen	Benzodiazepine	-
HDL Chol	1.1 mmol/L	1.2–1.7 mmol/L
Other Lipids	Normal	-
**Cerebrospinal fluid**		
CSF WCC	< 1	
CSF RBC	520	
CSF Lactate	1.45 mmol/L	1.1–2.4 mmol/L
CSF Protein	0.18 g/L	0.13–0.40 g/L
CSF Glucose	3.5 mmol/L	2.2–3.9 mmol/L
CSF Micro*scopy*	Negative	-
CSF Viral Screen *(Adenovirus, CMV, EBV, HSV 1 & 2, VZV, Enterovirus, Parechovirus, SARS-CoV-2)*	Negative	-
CSF Culture	Negative	-
**Other**		
Urine Microscopy	Negative	-
Urine Culture	Negative	-

a. Results outside the normal range. NMDA, *N*-methyl-d-aspartate receptor; CASPR2, contactin associated protein 2; LGI1, leucine-rich, glioma-inactivated 1; IL, interleukin; Anti Tr, anti-purkinje cell antibody; IgG, Immunoglobulin G; anti-Hu, type I anti-neuronal nuclear antibody; anti-Yo, anti-Purkinje cell antibody; anit-Ri, type II anti-neuronal nuclear antibody; TSH, thyroid-stimulating hormone; T4, thyroxine; B12, vitamin B12; HDL Chol, high-density lipoprotein cholesterol; CSF, cerebrospinal fluid; WCC, white cell count; RBC, red blood cell count; CMV, cytomegalovirus; EBV, Epstein–Barr virus; HSV, Herpes simplex virus; VZV, varicella zoster virus; SARS-CoV-2, severe acute respiratory syndrome, Coronavirus 2.

Early positive investigation results included a positive COVID-19 swab test, raised CRP, ferritin, D-dimer and TNF-alpha, characteristic of proinflammatory states. TNF-alpha remained elevated in the absence of other viral or bacterial causes. The positive test for benzodiazepines is noted secondary to lorazepam use alone.

Imaging demonstrated radiological changes consistent with COVID-19 on chest CT and chest X-ray, with no clear abnormality on magnetic resonance imaging (MRI) of the head. An electroencephalogram (EEG) revealed no evidence of seizures or encephalopathy.

On day 10 of her second admission she was oriented to month, year, day and place, but not date. She scored 58/100 on the Addenbrooke's Cognitive Examination-III with primary deficits in fluency and memory (attention 12/18, memory 7/26, fluency 4/14, language 23/26, visuo-spatial 12/16). She was perseverant at times, asking repetitively about when her spouse was visiting and how long she was likely to remain in hospital. She had pressured speech.

On day 14 she was switched from haloperidol to 0.5 mg risperidone and her delusional symptoms persisted.

On day 20 she was discharged at her request as psychotic symptoms were improving. She had paranoid thoughts but there was no evidence of confusion or disorientation. On day 29 it was noted during a follow-up call that she had stopped taking risperidone 2 days previously. She reported no auditory hallucinations but displayed tangential speech and further follow-up was arranged.

On day 52 she reported that for 2 weeks after discharge (up to day 34) she had experienced paranoid thoughts and had a feeling that she would be kidnapped. She had no cognitive deficits, a normal sleep cycle and her family noticed no changes in her functioning other than increased anxiety and hypervigilance of others when outside the house. She began to improve after this point and reported no psychotic symptoms by day 52.

## Discussion

The patient was admitted with psychotic symptoms including ongoing auditory hallucinations, a Capgras delusion and a complex systematised delusional misperception. These symptoms were consistent, regardless of fluctuating attention and followed COVID-19 infection. Initial symptoms were probably related to delirium but psychotic symptoms persisted as confusion improved. She responded to neuroleptic treatment and stopped taking it. Her psychotic symptoms persisted for around 3 weeks after the resolution delirium symptoms.

The patient was in good physical health prior to developing COVID-19 with no previous psychiatric history or symptoms to suggest a propensity towards psychiatric problems but we cannot rule out that the presentation was related to psychological stress. However, this appears unlikely given the onset of symptoms associated with an inflammatory state and no previous evidence of psychiatric symptoms while an in-patient in hospital during the first admission.

COVID-19 is associated with a proinflammatory cytokine state. In this case, the patient had raised levels of TNF-alpha, low lymphocyte count and a raised CRP in the absence of any cerebrospinal fluid evidence of virus or MRI and EEG evidence of an encephalopathy. Other antibodies associated with post-infectious changes in mental state such, as *N*-methyl-d-aspartate receptor, CASPR 2 and LGI1 were negative.

Although there is growing evidence of the neurological and psychiatric sequelae of COVID-19, this is the first detailed case report of psychotic symptoms that persisted following the acute phase of the illness and obvious delirium, and is not associated with the use of steroid treatment. In other coronavirus infections such as severe acute respiratory syndrome or Middle East respiratory syndrome, psychosis was reported in a small minority (0.7%) of patients and were primarily related to steroid use. During the Spanish influenza pandemic ‘psychoses of influenza’ were widely reported, and described by Karl Menninger in his case series of 100 patients with mental disturbances associated with influenza infection. He subsequently described two forms of post-influenza psychoses; schizophrenia deliriosa and delirium schizophrenoides,^12,13^ the latter ‘a psychosis arising in close association with the somatic illness but so colored with the hues and tints of schizophrenia that one feels obliged to give a pessimistic prognosis which is usually belied by the further course of the disease, namely, it's eventual disappearance’.

Epidemiological evidence has shown an increased risk of psychosis in people with a history of severe infection^14,15^ and genome-wide association studies have implicated multiple immune signalling pathways.^16^ Although it is difficult to unpick whether the raised inflammatory cytokine profile was causative of psychotic symptoms beyond a delirious period, raised TNF-alpha, as seen in this patient, is associated with first-episode psychosis and psychotic relapse.^17^ However, IL-6 elevation has also been implicated in first-episode psychosis and this was not observed in this case.^18,19^ Other potential mechanisms to consider are viral ‘proteiform’ disease, entering via the nasal passage initially, subsequently affecting the olfactory bulbs that are abundant in angiotensin-converting enzyme 2 receptors^20^ and directly invade the rest of the nervous system. This could lead to molecular mimicry where antibodies against the virus might cross-react with various epitopes in the nervous system.^5,21^

### Implications

In this case report we detailed the neurological investigations performed in a patient with persistent psychotic symptoms following a COVID-19 infection. We found an elevated TNF in the early course of the illness without a raised IL-6. In offering a cause for these symptoms we propose that elevated cytokines levels could be associated with a propensity to psychosis in some following a COVID-19 infection although we note that elevated cytokines are also associated with delirium. An alternative is a viral-induced antibody-mediated encephalitic event. Although this patient's case cannot confirm a causal link between COVID-19 infection and the development of psychosis, we propose that cytokine profiles are taken in patients with psychiatric symptoms in COVID-19 in addition to neurological investigations in order to help inform the management of psychotic symptoms in some patients.

## Data Availability

The authors have included relevant clinical data but not all clinical data are included in this published article. The full datasets are available from the corresponding author on reasonable request but patient confidentially is paramount.
